# Intoxication with nutmeg: A case presentation and analysis of Swiss poisons information center (Tox Info Suisse) enquiries 2014–2023

**DOI:** 10.1016/j.toxrep.2026.102274

**Published:** 2026-05-10

**Authors:** Amadea Bleisch, Selina Späni, Regina Schumann, Kanchan Dongre, Colette Degrandi, Anne Leuppi-Taegtmeyer

**Affiliations:** a Intensive Care Unit, Cantonal Hospital Baselland, Rheinstrasse 26, Liestal 4410, Switzerland; b Hospital Pharmacy, Cantonal Hospital Baselland, Rheinstrasse 26, Liestal 4410, Switzerland; c Department of Patient Safety, University Hospital Basel, Spitalstrasse 22, Basel 4031, Switzerland; d Tox Info Suisse, Affiliated Institute of the University of Zürich, Freiestrasse 16, Zürich 8032, Switzerland

**Keywords:** Myristica intoxication, Psychoactive agents, Social media, Poison Control Centers

## Abstract

**Introduction:**

Nutmeg is a widely used culinary spice. When ingested in large quantities it has psychoactive and hallucinogenic effects.

**Case reports:**

We present the case of a 23-year-old man on long-term fluoxetine and quetiapine who ingested 75 g of nutmeg with suicidal intent. Four hours later, he presented himself to the emergency department of the nearest hospital with tachycardia and mydriasis. He was transferred to the intensive care unit of Cantonal Hospital Baselland for further monitoring where, approximately 14 h after ingestion, he became somnolent. All symptoms resolved spontaneously within 26 h.

We also analyzed enquiries to the Swiss national poison information center (Tox Info Suisse) about possible nutmeg intoxication from 2014 to 2023. During this period, enquiries for 236 patients were made (51% female, 0 – 85 years old). The largest number of enquiries (N = 30) occurred during 2020 and 2021, coinciding with a TikTok nutmeg challenge and an increase in the popularity of home baking during the COVID-19 pandemic.

**Discussion:**

Nutmeg is a widely available household spice with ingredients that can cause toxic effects when ingested in large quantities. Intoxications usually show a mild course, as in the case we describe here, despite long term medication with fluoxetine. The analysis of enquires to Tox Info Suisse suggest that the incidence of enquiries about possible nutmeg intoxication might be subject to social influences.

## Highlights

• Large amounts of nutmeg can cause hallucinations, tachycardia and drowsiness.
• Nutmeg intoxication is treated symptomatically, with symptoms lasting 24 – 72 h.
• Swiss poison center data show mostly mild nutmeg intoxications from 2014 to 2023.
• Poison center data suggest social influences may affect nutmeg intoxication rates.

## Graphical Abstract

**Figure:**
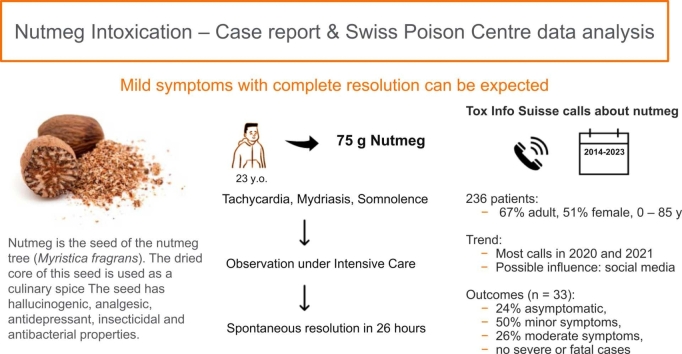

## Background

Nutmeg is the seed of the nutmeg tree (*Myristica fragrans*). The dried core of this seed is used as a culinary spice and is widely available in most households. The seed has hallucinogenic, analgesic, antidepressant, insecticidal and antibacterial properties [1]. When ingested in large quantities, nutmeg has similar effects to MDMA (“ecstasy”) and so also has potential for abuse [1]. Accidental overdoses, deliberate ingestion on account of its hallucinogenic properties and ingestion with suicidal intent have been reported [2]. In 2020, a TikTok Challenge led to nutmeg intoxications in teenagers [3]. In this challenge participants ingest a large amount of nutmeg to achieve a 'natural high'. This is shared in a 15 s video that inspires others to do the same.

## Case presentation

We present the case of a 23-year-old man with a current major depressive episode who ingested 75 g of ground nutmeg with suicidal intent and self-presented to the nearest emergency department (ED). The amount of nutmeg ingested was self-reported and he denied co-ingestion of other substances. He had a medical history of recurrent depressive disorder, Asperger's syndrome, gender dysphoria, panic disorder and obsessive-compulsive disorder and was taking fluoxetine 20 mg twice daily and quetiapine 150 mg once daily long-term.

When the patient was examined in the ED four hours after ingesting nutmeg, he was very taciturn but oriented in time and place and symptom-free. He denied hallucinations. Vital signs showed a sinus tachycardia of 140 beats per minute with a blood pressure of 129/59 mmHg and an oxygen saturation of 97% breathing room air. Physical examination additionally revealed bilateral mydriasis. These initial findings were interpreted as resulting from mild anticholinergic symptoms. Routine blood tests showed no relevant abnormalities.

The patient was transferred to the intensive care unit of Cantonal Hospital Baselland for monitoring. On arrival there (approximately 14 h after ingestion), he presented with a Glasgow Coma Scale (GCS) score of 13 as he was somnolent and disoriented to time, which normalized after a few hours. He had a pronounced startle response, which was interpreted in the context of his psychiatric diagnoses. The heart rate normalized spontaneously 26 h after ingestion and blood pressure remained normal throughout. No symptomatic therapy was necessary, and the patient could be discharged to a psychiatric hospital 30 h after admission. The patient gave their written informed consent for the publication of this case in accordance with the Declaration of Helsinki.

### Case and time series analysis of enquiries to Tox Info Suisse

**Fig. 1:**
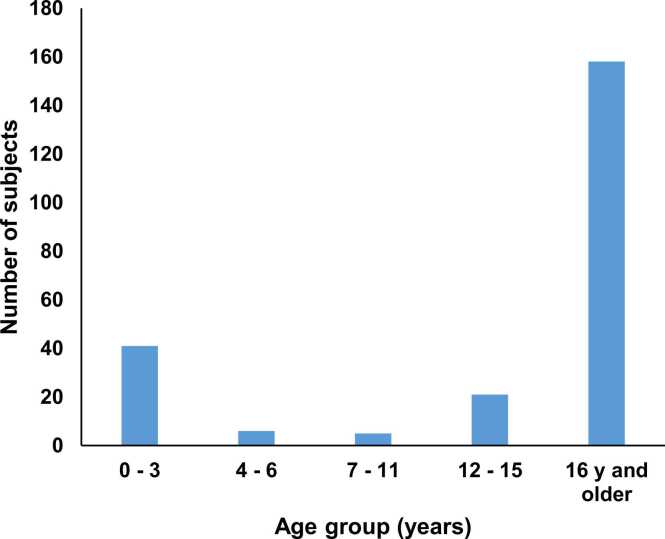
Number of enquiries to Tox Info Suisse regarding nutmeg by subject age group 2014 – 2023 (N = 231, the exact ages of five subjects under 16-years old were unknown so these were excluded).

After managing this case, we were interested to gain more information about nutmeg intoxication in Switzerland. To these ends, we were granted ethical committee approval to examine anonymous Tox Info Suisse reports (EKNZ BASEC Req-2025–01574)”. Tox Info Suisse received enquiries for 236 patients (51% female) regarding ingestion of nutmeg as a single substance between 2014 and 2023. There were 78 children (< 16 years old) and 158 adults (16 years or older) (Fig. 1).

**Fig. 2:**
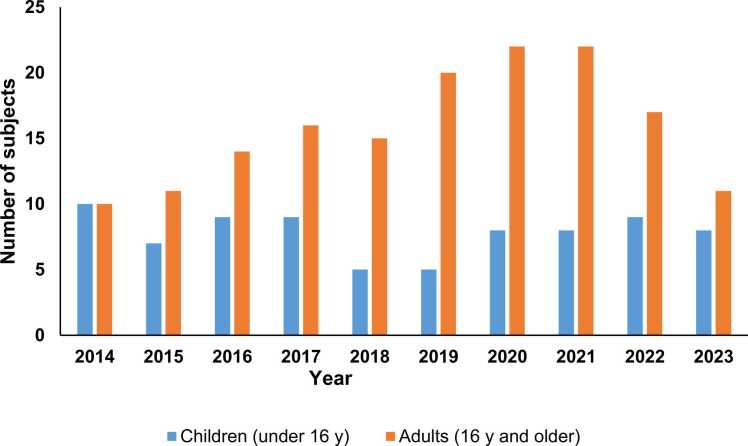
Number of enquiries to Tox Info Suisse regarding nutmeg by subject age group and year 2014 – 2023 (N = 236).

**Fig. 3:**
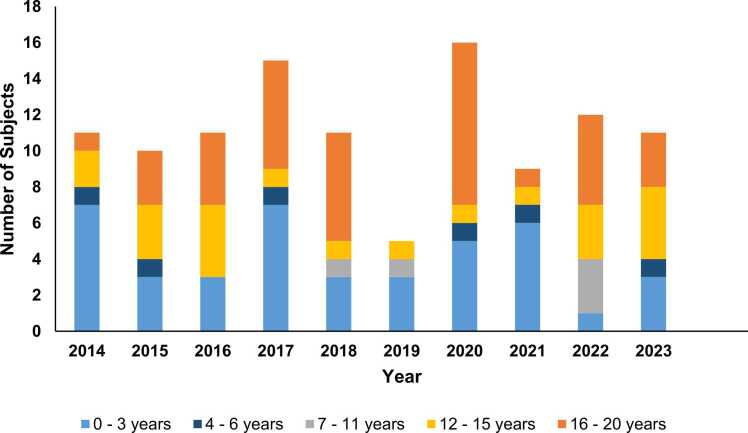
Number of enquiries to Tox Info Suisse regarding nutmeg in children and young adults 2014 – 2023 (N = 111 for whom age available).

The exact age in years was known for 73 children and for 105 adults and ranged from 0 to 85 years. Most enquiries about children were for infants aged 0 – 3 years (Fig. 1). Fig. 2 shows the distribution of enquiries for adults compared to children over time (Fig. 2) and Fig. 3 shows the time course of enquiries for subjects up until the age of 20 years for whom the exact age was known (Fig. 3).

We did not perform an age-specific analysis for adults on account of the large amount of missing data (age was not available in 53 cases) and potential for bias. The largest number of enquiries (N = 30) in the studied ten-year period were during 2020 and 2021.

Written documentation regarding symptoms and medical follow-up was available for seven children and 26 adults. Symptom severity was graded according to the Poisoning Severity Score (PSS) [4]. Twenty-four percent of cases (N = 8) were asymptomatic, of which 50% were adult and 50% were children. Minor symptoms (as in the case reported here) were reported in 48% (N = 16), of which 87.5% were adults and 12.5% were children. Moderate symptoms were reported in the remaining 27% (N = 9), of which 89% were adults and 11% were children. Moderate symptoms included tachycardia (heart rate > 140 beats per minute) and neurological symptoms such as stupor, agitation, hallucinations or myoclonus. There were no severe or fatal intoxications. Data about ingested nutmeg doses were available in 18 cases with medical follow-up. Median ingested doses were 10 g among asymptomatic cases (N = 2, range 5 – 15 g), 18.25 g among cases with mild symptoms (N = 10, range 10 – 132 g) and 22.5 g among cases with moderate symptoms (N = 6, range 15 – 50 g).

## Discussion

In this report, we describe both a clinical case and a ten-year case series of intoxications with nutmeg in Switzerland. The largest number of enquiries to Tox Info Suisse about nutmeg ingestions occurred in 2020 and 2021. Reasons for this could be a TikTok nutmeg consumption challenge launched in 2020 [3] and the heightened popularity of home baking during the COVID-19 pandemic [5].

The pharmacological properties of nutmeg are attributed to myristicin, safrole and elemicin [6]. There is circumstantial evidence that myristicin is metabolized to amphetamine-like substances such as MMDA (3-methoxy −4,5 methylenedioxyamphetamine) [1], an analog of MDMA (3,4 methylenedioxymethamphetamine), which is primarily responsible for nutmeg's hallucinogenic effects [7], [8]. However, animal and human studies of urine from subjects who have ingested large quantities of nutmeg could not confirm this [9], [10]. MDMA has a stimulating effect on the 5-HT_2A_ receptor and increases the release of serotonin, noradrenaline and dopamine [6]. Based on theoretical considerations, a pharmacodynamic interaction with the SSRI fluoxetine could be possible. Safroles are also precursors of MDMA and MDA (3,4-methylendioxyamphetamin). Elemicin and myristicin have anticholinergic properties [2]. Myristicin is also a weak monoamine oxidase inhibitor that slows down the breakdown of serotonin, noradrenaline and dopamine [1]. Cardiovascular reactions such as tachycardia can be explained by the serotoninergic effects of nutmeg’s ingredients as well as increased noradrenergic activity secondary to slowed neurotransmitter degradation caused by monoamine oxidase inhibition [1].

Features of intoxication include euphoria, hallucination, agitation, delirium, CNS depression, anticholinergic syndrome, tachycardia, headache, nausea, vomiting, mydriasis or miosis [6], [11]. Neuropsychological symptoms can be expected after ingestion of 10–15 g of nutmeg, and anticholinergic effects are likely above 25–28 g [1]. Lethal courses in adults have not yet been described for single nutmeg ingestions below 80 g without co-ingestion of other substances in overdose [12]. After ingestion, symptoms occur within one to six hours and usually last no longer than 24 h, with a maximum duration of 72 h [1], [6]. When large amounts of nutmeg are ingested in combination with selective serotonin reuptake inhibitors, an additive risk of developing serotonergic toxicity seems plausible. A case series from Illinois also reported that patients who coingested nutmeg and psychoactive medication showed more serious toxicity [2].

The treatment of nutmeg intoxication depends on the symptoms [11]. Benzodiazepines can be administered for agitation. If coma develops as part of a severe central anticholinergic syndrome, treatment with physiostigmine, a cholinesterase inhibitor, can be considered, however its use is controversial [13]. In case of overdose, symptoms can persist over several hours as the half-lives of myristicin, safrole and elemicin are 19, 17 and 8.5 h, respectively [14]. According to the available case reports and studies, a mild course can be expected in most cases of intoxication [2], [11], [14].

## Conclusions

Nutmeg is a widely available household spice with ingredients that can cause toxic effects when ingested in large quantities. Intoxications usually show a mild course, as in the case we describe here, despite long term medication with fluoxetine. A case and time series analysis of enquiries related to nutmeg intoxication received by the Swiss national poisons center indicates that the incidence of such intoxications might be influenced by social factors such as the nutmeg challenge in social media and the rise in home baking trends observed during the COVID-19 pandemic.

## CRediT authorship contribution statement

**Kanchan Dongre:** Visualization, Formal analysis. **Colette Degrandi:** Writing – review & editing, Supervision, Methodology, Data curation. **Anne Leuppi-Taegtmeyer:** Writing – review & editing, Supervision, Project administration, Methodology, Formal analysis, Data curation, Conceptualization. **Amadea Bleisch:** Writing – original draft, Project administration, Methodology, Formal analysis, Conceptualization. **Selina Späni:** Writing – review & editing, Writing – original draft, Methodology. **Regina Schumann:** Writing – review & editing, Formal analysis, Data curation.

## Declaration of Competing Interest

None.

## Data Availability

Data will be made available on request.
